# Coexistent Sjogren’s syndrome and Birt-Hogg-Dube´ syndrome: a case report

**DOI:** 10.1186/s12890-023-02680-5

**Published:** 2023-11-22

**Authors:** Yongkang Lin, Ting Guo, Cheng Lei, Binyi Yang, Danhui Yang, Hong Luo, Hong Peng

**Affiliations:** 1 Department of Pulmonary and Critical Care Medicine, the Second Xiangya Hospital, Central South University, 139 Renmin Middle Road, Furong District, Changsha, Hunan 410011 China; 2 Research Unit of Respiratory Disease, Central South University, Changsha, Hunan 410011 China; 3 Hunan Diagnosis and Treatment Center of Respiratory Disease, Changsha, Hunan 410011 China

**Keywords:** Sjogren’s syndrome, Birt-Hogg-Dubé syndrome, DCLD, FLCN, Gene, Diagnostic algorithm

## Abstract

We report a rare case of Sjogren’s syndrome complicated with Birt-Hogg-Dubé syndrome (BHDS) not previously mentioned in the literature. Further, there is insufficient evidence linking the two diseases. Here, we review existing diagnostic algorithms for diagnosing diffuse cystic lung disease and provide new insights. The patient initially complained of thirst and dry eyes for ten years, and gradually developed shortness of breath. After admission, physical examination showed five missing teeth, decreased respiratory sounds in both lower lungs, and Velcro rales. Computed tomography showed multiple thin-walled cystic lesions in both lungs. Initial xerophthalmia and labial gland biopsy seemed to reveal a pulmonary cystic change associated with Sjogren’s syndrome. Before discharge, a rash suspected to indicate a fibrofollicular tumor in the neck was observed, and then *FLCN* variant has been found. The challenges how to clarify the diagnosis of DCLD causes are discussed.

## Introduction

Diffuse cystic lung disease (DCLD) is a group of heterogeneous diseases with different pathophysiological mechanisms involving the production of many spherical or irregular thin-walled inflatable spaces in the lung parenchyma. However, because of the many causes of DCLD (including pulmonary Langerhans cell histiocytosis and lymphangioleiomyomatosis) and its nonspecific manifestation, diagnosing the cause of the disease is relatively difficult. Fortunately, some progress has been made, including the creation of a series of diagnostic algorithms; however, they are complicated and often involve invasive lung biopsy [1–3].

Birt-Hogg-Dubé syndrome (BHDS, MIM 135,150), or Hornstein-Knickenberg syndrome, is an autosomal dominant hereditary disorder associated with a germline pathogenic variant *folliculin* (*FLCN*), which increases risk of benign cutaneous fibrofolliculomas, pulmonary cysts, spontaneous pneumothorax, and multiple, mainly malignant, bilateral and multifocal renal neoplasias. More than 80% of those with BHDS with lung involvement have pulmonary cysts, and DCLD and spontaneous pneumothorax can occur [4]. Among an East Asian BHDS cohort, the incidence of multiple pulmonary cysts was 87.3% (100/162), with 74.7% (124/162) of patients having a history of at least one pneumothorax [5]. A literature review of studies performed in China revealed that surrounding multiple pulmonary cysts was higher (195/221) than the Caucasians, and family history of pneumothorax was 84.7% [6].

Sjogren’s syndrome (SS) is an autoimmune disorder characterized by lymphocytic infiltration of salivary and lachrymal glands, leading to sicca syndrome. Although subclinical lung abnormalities occur in > 50% of patients, clinically meaningful lung involvement affects only about 10-20% [7–10]. The mechanism of SS-associated pulmonary cystic degeneration is unknown. However, discrepancies exist in the literature concerning the frequency and clinical significance of cystic lung disease in patients with SS. In one study, 90 patients with SS followed up at the department of internal medicine underwent a systematic chest computed tomography (CT) scan, revealing twenty-one (23.3%) with lung cysts [11].

No literature reports of the coexistence of BHDs with SS are available. Since there is no effective treatment for DCLD, it is very important to accurately diagnose the etiology of DCLD. We share a case of BHDs with Sjogren’s syndrome and provide new insights into the diagnostic process.

## Clinical report

**Fig. 1:**
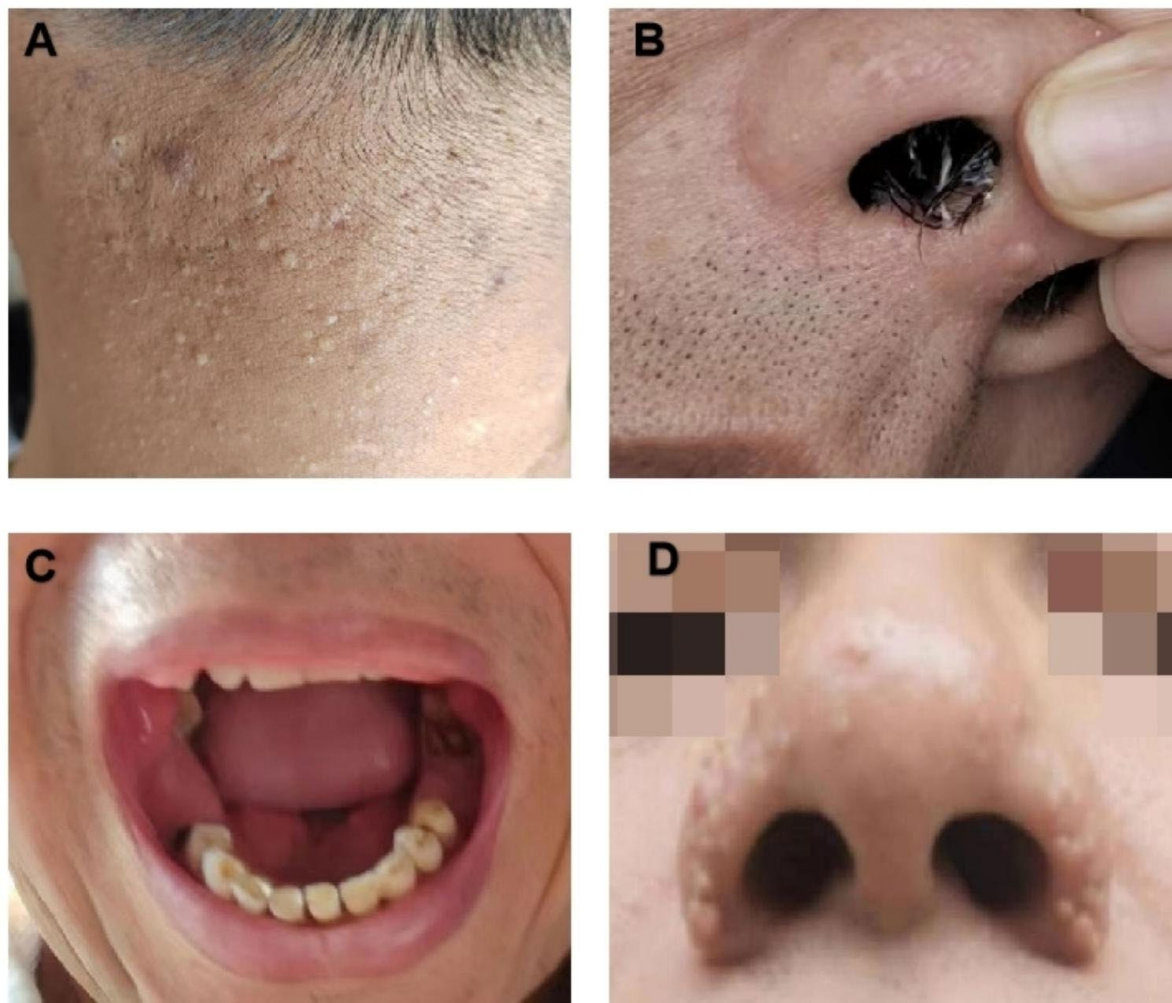
Physical examination findings are shown. **(A-B)** The proband had normochromic papules in the back of his neck and in his nose. **(C)** He had lost five teeth successively due to caries in the last three years, with several dental caries remaining. **(D)** His brother’s normochromic nose papules are shown

**Fig. 2:**
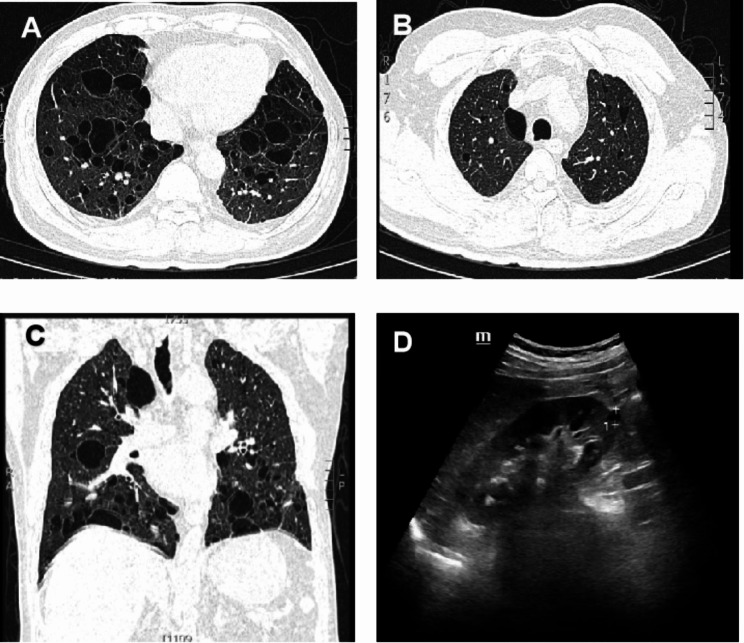
Imaging findings. **(A-B)** Lung HRCT shows multiple thin-walled cystic lesions throughout the lung bilateral diffuse thin walled cystic changes with a wide variation in cyst size and distribution (the biggest one > 50 mm). **(C)** Frontal plane reconstruction showing multiple cysts adjacent to blood vessels, fissures and visceral pleura, with predominantly basal and paramediastinal location. **(D)** Abdominal ultrasound indicates a right renal cyst

**Fig. 3:**
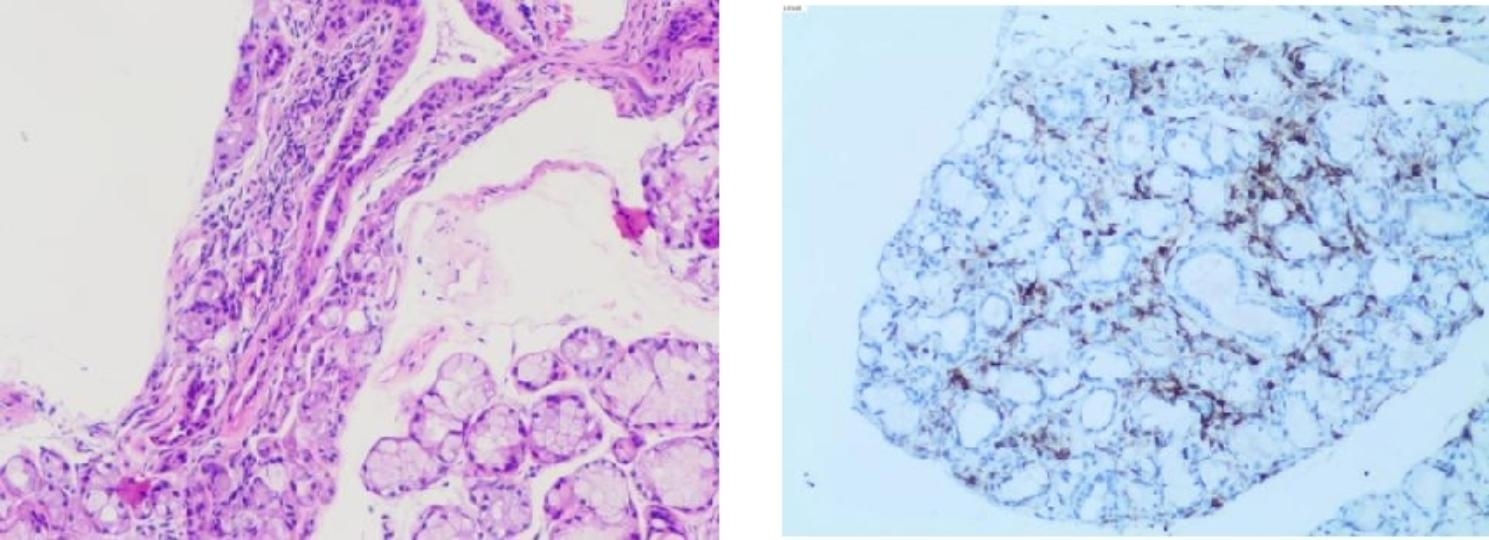
Left lower lip gland biopsy. Salivary gland tissue structure was normal, with interstitial foci infiltration into increased lymphocytes (about 50/focus) revealed via immunohistochemistry: LCA (+). These are typical pathological manifestations of Sjögren’s syndrome

A 55-year-old man from Pingxiang City, Jiangxi Province, China, complained of thirst and dry eyes for the last 10 years, and repeated cough, expectoration, and shortness of breath for the last 7 years. In 2015, he was diagnosed with ‘chronic obstructive pulmonary disease’ and treated with tiotropium bromide inhalation. From 2016 to 2020, CT showed multiple thin-walled cystic lesions in both lungs. Lung function tests showed FEV1/FVC:74.02% and FEV1/pred:69.0% in 2020. In April 2022, the patient developed a cough after catching a cold, yellow-green phlegm, and shortness of breath after strenuous activity. He had obvious symptoms of dry mouth, thirst, and dry eyes throughout the past ten years, reportedly drinking about 3–4 L of water daily, even waking at 3–4 am for water due to thirst and occasionally using drops to relieve eye dryness. On physical examination, he said he had tooth loss in the last three years (Fig. 1), and we found several dental caries. In the lower lungs, Velcro rales were heard. Pulmonary function tests revealed mild obstructive pulmonary ventilation dysfunction with small airway dysfunction (FEV1/FVC:78.13%, FEV1/pred:77.6%). Lung CT showed multiple thin-walled cystic lesions in both lungs (Fig. 2). Abdominal ultrasound indicated a right renal cyst. Left lower lip gland biopsy revealed nearly normal salivary gland tissue structure, with interstitial foci infiltration with increased lymphocytes (about 50/focus) (Fig. 3). Tear film breakup time was abnormal. Schirmer test results showed the following: right eye, 0 mm; left eye, 0 mm. Immunologic examination showed: ANA (-), ENA (-), anti-SSA(60 KD)(-), anti-Ro antibody (52 KD) (-), ANCA (-). Using international diagnostic standard for SS in 2016 criteria, the patient scored > 4 points [12]. SS was considered after a consultation with the rheumatic immunology department. Our senior doctor observed normochromic papules in the back of the neck suspected to be fibrofolliculoma. Although the patient had no family history of pneumothorax, we completed genetic testing to rule out BHDS prior to hospital discharge.

**Fig. 4:**
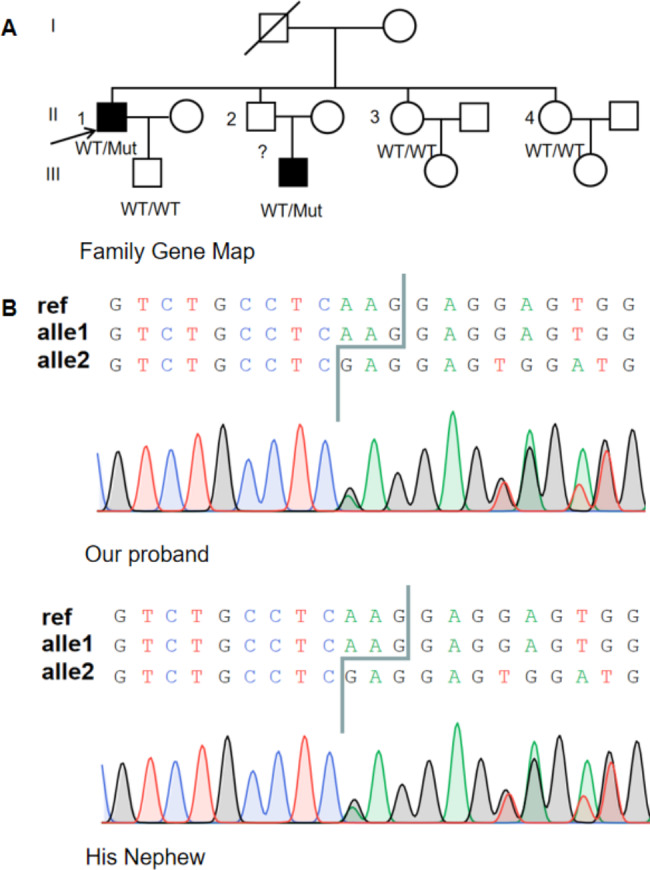
Pedigree of the proband and Sanger sequencing. **(A)** A Pedigree of the proband is shown. **(B)** Sanger sequencing findings of the proband and his nephew revealing a heterozygous *FLCN* variant (NM_144997.7:c.1522_1524del, p.(Lys508del)) are shown

Genomic DNA of the patient and his relatives was isolated using a QIAamp DNA Blood Mini Kit in accordance with the manufacturer’s instructions. cDNA of *FLCN* was amplified using GoldenStar® T6 Polymerase (TSE101, Tsingke), with primers designed using the NCBI primer blast tool (https://www.ncbi.nlm.nih.gov/tools/primer-blast/). Polymerase chain reaction (PCR) and Sanger sequencing identified an *FLCN* variant (NM_144997.7: c.1522_1524del p.(Lys508del)) (Fig. 4). The variant was considered potentially pathogenic via ClinVar (www.ncbi.nlm.nih.gov/clinvar/) and the variant site was reported previously [13]. No variants were in the other thirteen exons of *FLCN*. Therefore, the patient had BHDS according to 2009 diagnostic criteria. A patient with the same variant was previously reported to have fibrofolliculomata and family history of spontaneous pneumothorax but no renal tumor [13]. Family analysis showed that although the proband’s younger brother did not undergo genetic testing and lung HRCT, he likely has the same variant because he had a typical rash on the nose and his son had the variant. We believe that the proband’s brother and nephew fulfill the diagnostic criteria of BHDS. Other family members had no variant (Fig. 4). A discussion of multidisciplinary treatment (MDT) regarding the above comprehensive tests prompted a diagnosis of BHDS complicated with SS.

## Discussion and conclusion

Here, we report a patient with DCLD as a primary disease manifestation who was initially diagnosed SS and later with SS with BHDS. For patients with DCLD, the treatment of the cause is indispensable. However, its cause may be diverse, multiple underlying diseases are possible. Although scholars summarize some diagnostic algorithms, still may lead to missed diagnosis [1, 2]. Therefore, we provide new insights regarding the previous diagnostic algorithm used.

HRCT characteristic analysis is a good diagnostic model, with literature suggesting that HRCT can be definitively diagnosed in 80% of patients with DCLD [14]. In 2016, researchers proposed an imaging-based diagnostic algorithm [2]; however, BHDS and SS can not be distinguished by pulmonary HRCT alone. It stated that there were more cysts at the base of the lung or adjacent to mediastinum blood vessel in BHDS, while the patients with SS had random distribution and large size heterogeneity [1]. Our patient has cysts located at the base of the lung and adjacent to mediastinum blood vessels but other lobes are also involved. Therefore, it is difficult to achieve an accurate diagnosis via the use of an imaging-based diagnostic algorithm.

Another diagnostic from China is based initially on a patient’s complete medical history data and meaningful examinations, and finally involves genetic and pathological examinations [3]. There was no doubt that our patient met diagnostic criteria of SS; therefore, we initially thought that it was the sole DCLD cause. However, it is worth noting that the patient also met diagnostic criteria for BHDS, although he had no family history of pneumothorax nor did other offspring lacked lung symptoms or DCLD. This diagnosis algorithm does not pay enough attention to DCLD patients with multiple causes. We consider that the etiological diagnosis should be as perfect as possible, because when the source of DCLD can not be excluded, the lack of multi-etiological secondary prevention is likely to affect the prognosis of patients.

Main causes of DCLD include LAM, Birt–Hogg–Dubé syndrome, pulmonary Langerhans cell histiocytosis, lymphoid interstitial pneumonia (including primary and secondary), amyloidosis, light-chain deposition disease, Sjögren syndrome, and primary or metastatic neoplasm. Since our patient has characteristic clinical features and investigations fitting into diagnostic criteria of Sjogren’s syndrome, a diagnosis of cystic lung disease secondary to Sjogren syndrome was entertained. However, secondary LIP, which is common in some rheumatic diseases, such as rheumatoid arthritis and Sjogren’s syndrome, cannot be excluded. Lung biopsy is required for diagnosis. Because the patient worried the high cost and trauma, so the surgical biopsy was refused. At the same time, we considered even if the biopsy was completed, it would not helpful to the treatment. Lung cysts in LIP are typically small (< 3 cm), and usually located within areas of GGOs. The presence of ancillary parenchymal abnormalities, including bilateral GGOs and poorly defined centrilobular nodules, frequently in a subpleural distribution, can reinforce the diagnosis of LIP but this case is characterized by a simple cyst in the lung [15]. At the same time, Spontaneous pneumothorax has also been reported in DCLD secondary to Sjögren syndrome and lymphoid interstitial pneumonia but less than BHDS and LAM [16]. SS and BHDS have similar changes in the pulmonary function changes in long term and imaging features [1, 11]. Our proband was diagnosed with ‘chronic obstructive pulmonary disease’ and underwent long-term pulmonary function monitoring, with no significant decline in lung function observed. This is consistent with the description of such patients in the literature. Since symptoms of SS-related pulmonary cystic degeneration are not obvious, treatment is mainly confined to the treatment of the primary disease.

BHDS is an autosomal dominant disorder. As the only gene known to underly BHDS, many pathogenic variants have been identified in every coding exon of *FLCN*. The *FLCN* variant c.1285dupC/delC in exon 11 has been most frequently observed in Asian patients [5]. Here, a germline heterozygous variant of *FLCN* was found, which corresponded to a deletion of three bases (1522–1524) *FLCN* bases, resulting in an mRNA without lysine 508 (delK508). This variant decreases FLCN protein levels, leading to cystic degeneration. Patients with variants in exon 12 have a higher incidence of pneumothorax versus those with variants in other exons. Furthermore, those with variants in exon 9 had more cysts [17] and those with variants in exons 9 and 12 had the largest cyst diameter and volume [17]. Patients with these variants should be warned that they may develop pneumothorax in the future [18]. The variant site of our patient was not in exons 9 or 12. However, our proband had a substantial number of large pulmonary lung cysts; therefore, variants at this locus may indicate severe lung imaging changes. Alternatively, this manifestation may be unique to disease due to BHDS and SS. However, additional clinical data for validation are needed.

Lung cysts usually appear between the ages of 40 and 50 years in those with BHDS [19]. Our proband reported that since pulmonary cystic lesions were accidentally discovered in his early twenties, no pneumothorax occurred. In contrast, > 80% East Asian patients have had a family history of spontaneous pneumothorax. Further, the presence, number, size, and total volume of lung cysts and family history of pneumothorax have been identified as risk factors for developing pneumothorax in BHDS [17]. Notably, the current patient had a large number of pulmonary cysts in both lungs, with a maximum size of about 40 mm. In previous literature, no gender difference was found between Europeans and Americans. However, our literature analysis revealed that East Asians and Europeans and Americans with the same variant tend to have different clinical manifestations [5]. In studies in which this variant has been reported, patients typically had a familial history of pneumothorax and were all female. Lung cysts in patients with BHDS are mostly caused by loss-of-function *FLCN* variants. At present, there seems to be no special treatment for BHDS-related cystic lung disease. Lung involvement is limited to the prevention and treatment of pneumothorax. According to the previous literature, pleurodesis is recommended after the first attack of spontaneous pneumothorax [20]. There is currently no evidence of a clear association between renal cysts and renal malignancies. Because BHDS is highly likely associated with renal malignancy which is an indicator of poor prognosis, we recommend annual renal imaging in carriers. Skin involvement is common in Caucasians but less common in Asians [1, 17, 21], with fibrofolliculoma a characteristic lesion. The proband and his younger brother exhibited the typical rash. Surgical rash excision is performed only when necessary due to the low probability of transformation to a malignant lesion.

In conclusion, we report a patient with DCLD who was finally diagnosed with BHDS complicated with SS. According to the previous diagnostic process, when patients with diffuse pulmonary cystic lesions are encountered, based on general conditions and comprehensive auxiliary inspection, a relatively accurate diagnosis can be obtained. However, we propose that multiple causes of DCLD may be present the same patient and cause similar manifestations. Therefore, when all the symptoms or manifestations of the patient cannot be explained using a “monistic” diagnostic theory, a “dualistic” theory may be warranted. Multiple factors and diseases may cause lung cystic lesions, all with different treatments and prognoses. However, additional clinical data is needed. We compared our patient with those with BHDS caused by the same variant or DCLD combined with simple SS. We describe clinical manifestations derived from the overlap of both diseases for the first time to help guide the future diagnosis and treatment of DCLD.

## Data Availability

The datasets for this article are not publicly available due to concerns regarding participant/patient anonymity. Requests to access the datasets should be directed to the corresponding authors.

## References

[1] Gupta N, Vassallo R, Wikenheiser-Brokamp KA, McCormack FX (2015). Diffuse cystic lung disease. Part II. Am J Respir Crit Care Med.

[2] Raoof S, Bondalapati P, Vydyula R (2016). Cystic lung Diseases: Algorithmic Approach. Chest.

[3] Xu KF, Feng R, Cui H (2016). Diffuse cystic lung Diseases: diagnostic considerations. Semin Respir Crit Care Med.

[4] Daccord C, Good JM, Morren MA, Bonny O, Hohl D, Lazor R (2020). Birt-Hogg-Dubé syndrome. Eur Respir Rev.

[5] Guo T, Shen Q, Ouyang R (2020). The clinical characteristics of east asian patients with Birt-Hogg-Dubé syndrome. Ann Transl Med.

[6] Hu X, Zhang G, Chen X, Xu KF. Birt-Hogg-Dubé syndrome in Chinese patients: a literature review of 120 families. Orphanet J Rare Dis. 2021;16(1):223. Published 2021 May 17. 10.1186/s13023-021-01848-8. 10.1186/s13023-021-01848-8 PMC8130425 34001170

[7] Franquet T, Gimenez A, Monill JM (1997). Primary Sjögren syndrome and associated lung disease: CTfindings in 50 patients. AJR Am J Roentgenol.

[8] Ramos-Casals M, Solans R, Rosas J et al. Primary Sjögren syndrome in Spain: clinical and immunologic expression in 1010 patients. Med (Baltim) 200887:210–9. 10.1097/MD.0b013e318181e6af 18626304

[9] Nannini C, Jebakumar AJ, Crowson CS et al. Primary Sjögren syndrome 1976–2005 and associated interstitial lung disease: a population-based study of incidence and mortality. BMJ Open 20133:e003569. 10.1136/bmjopen-2013-003569 PMC3845035 24282246

[10] Ramos-Casals M, Brito-Zeron P, Solans R (2014). Systemic involvement in primary Sjögren syndrome evaluated by the EULAR-SS disease activity index:analysis of 921 spanish patients (GEAS-SS Registry). Rheumatology (Oxford).

[11] Lechtman S, Debray MP, Crestani B (2017). Cystic lung disease in Sjögren’s syndrome: an observational study. Joint Bone Spine.

[12] Shiboski CH, Shiboski SC, Seror R (2017).

[13] So SY (2009). Spontaneous pneumothorax due to Birt-Hogg-Dube syndrome in a chinese family. Respirology.

[14] Gupta N, Meraj R, Tanase D (2015). Accuracy of chest high-resolution computed tomography in diagnosing diffuse cystic lung diseases. Eur Respir J.

[15] Swigris JJ, Berry GJ, Raffin TA, Kuschner WG (2002). Lymphoid interstitial pneumonia: a narrative review. Chest.

[16] Gupta N, Wikenheiser-Brokamp KA, Fischer A, McCormack FX (2016). Diffuse cystic lung disease as the presenting manifestation of Sjögren Syndrome. Ann Am Thorac Soc.

[17] Toro JR, Pautler SE, Stewart L (2007). Lung cysts, spontaneous pneumothorax, and genetic associations in 89 families with Birt–Hogg–Dubé syndrome. Am J Respir Crit Care Med.

[18] Johannesma PC, van de Beek I, van der Wel JW (2016). Risk of spontaneous pneumothorax due to air travel and diving in patients with Birt-Hogg-Dubé syndrome. Springerplus.

[19] Kunogi M, Kurihara M, Ikegami TS (2010). Clinical and genetic spectrum of Birt–Hogg–Dubé syndrome patients in who m pneumothorax and/or multiple lung cysts are th02.02 e presenting feature. J Med Genet.

[20] Gupta N, Seyama K, McCormack FX (2013). Pulmonary manifestations of Birt–Hogg–Dubé syndrome. Fam Cancer.

[21] Schmidt LS, Linehan WM (2015). Molecular genetics and clinical features of Birt–Hogg–Dubé syndrome. Nat Rev Urol.

